# Does Viral Co-Infection Influence the Severity of Acute Respiratory Infection in Children?

**DOI:** 10.1371/journal.pone.0152481

**Published:** 2016-04-20

**Authors:** Miriam Cebey-López, Jethro Herberg, Jacobo Pardo-Seco, Alberto Gómez-Carballa, Nazareth Martinón-Torres, Antonio Salas, José María Martinón-Sánchez, Antonio Justicia, Irene Rivero-Calle, Edward Sumner, Colin Fink, Federico Martinón-Torres, GENDRES network

**Affiliations:** 1 Grupo de Investigación en Genética, Vacunas, Infecciones y Pediatría, Hospital Clínico Universitario and Universidade de Santiago de Compostela (USC), Galicia, Spain; 2 Translational Pediatrics and Infectious Diseases section, Department of Pediatrics, Hospital Clínico Universitario de Santiago, Santiago de Compostela, Galicia, Spain; 3 Section of Paediatrics, Division of Infectious Disease, Imperial College of London, South Kensington Campus, London, United Kingdom; 4 Unidade de Xenética, Departamento de Anatomía Patolóxica e Ciencias Forenses, and Instituto de Ciencias Forenses, Grupo de Medicina Xenómica (GMX), Facultade de Medicina, Universidade de Santiago de Compostela, Galicia, Spain; 5 Micropathology Ltd., University of Warwick Science Park, Coventry, United Kingdom; Kliniken der Stadt Köln gGmbH, GERMANY

## Abstract

**Background:**

Multiple viruses are often detected in children with respiratory infection but the significance of co-infection in pathogenesis, severity and outcome is unclear.

**Objectives:**

To correlate the presence of viral co-infection with clinical phenotype in children admitted with acute respiratory infections (ARI).

**Methods:**

We collected detailed clinical information on severity for children admitted with ARI as part of a Spanish prospective multicenter study (GENDRES network) between 2011–2013. A nested polymerase chain reaction (PCR) approach was used to detect respiratory viruses in respiratory secretions. Findings were compared to an independent cohort collected in the UK.

**Results:**

204 children were recruited in the main cohort and 97 in the replication cohort. The number of detected viruses did not correlate with any markers of severity. However, bacterial superinfection was associated with increased severity (OR: 4.356; *P*-value = 0.005), PICU admission (OR: 3.342; *P*-value = 0.006), higher clinical score (1.988; *P*-value = 0.002) respiratory support requirement (OR: 7.484; *P*-value < 0.001) and longer hospital length of stay (OR: 1.468; *P*-value < 0.001). In addition, pneumococcal vaccination was found to be a protective factor in terms of degree of respiratory distress (OR: 2.917; *P*-value = 0.035), PICU admission (OR: 0.301; *P*-value = 0.011), lower clinical score (-1.499; *P*-value = 0.021) respiratory support requirement (OR: 0.324; *P*-value = 0.016) and oxygen necessity (OR: 0.328; *P*-value = 0.001). All these findings were replicated in the UK cohort.

**Conclusion:**

The presence of more than one virus in hospitalized children with ARI is very frequent but it does not seem to have a major clinical impact in terms of severity. However bacterial superinfection increases the severity of the disease course. On the contrary, pneumococcal vaccination plays a protective role.

## Introduction

Molecular techniques including polymerase chain reaction (PCR) have increased the sensitivity of detection for common and emerging respiratory viruses, and often reveal the presence of more than one pathogen in respiratory patients.[1]^,^[2] The importance of viral co-infections in the pathogenesis, severity or course of respiratory infections is not well established. The high sensitivity of molecular techniques raises questions about the clinical relevance of positive test results. The presence of a virus does not necessarily indicate causation of clinical symptoms or disease [3]. On the contrary, bacterial co-infection is usually associated with a more severe diseases course and worse prognosis, despite the precise interaction between bacteria and viruses is not always clear.

Our study aims to analyze the relationship between viral or bacterial co-infection detected by molecular methods, and the clinical phenotype of children admitted to hospital with lower tract acute respiratory infections (LT-ARI).

## Materials and Methods

### Study design and recruitment criteria

Two independent observational prospective patient groups were enrolled in Spain (main group) and in the United Kingdom (replication group). Spanish children were recruited between January 2011 and January 2013 through a national hospital based research network: GENDRES (Genetic, vitamin D and Respiratory infections research network– www.gendres.org), which includes 13 Spanish tertiary hospitals. UK children were recruited between October 2009 and May 2010 at St Mary’s hospital (UK). In both cohorts, eligible study participants were children under 14 years of age with a lower respiratory tract illness of sufficient severity to warrant admission to hospital, and a virus identified on nasopharyngeal swab or aspirate sample. All types of LT-ARI were included, from bronchiolitis to pneumonia, with or without wheezing, fever, rhinorrhea and respiratory distress.

A nasopharyngeal sample (aspirate or swab) was obtained during admission for viral pathogen detection. Besides the diagnostic procedures performed at origin in the referring hospital, viral detection was performed in all recruited subjects using a panel of 19 viruses, by nested PCR: respiratory syncytial virus; influenza virus (A, B); parainfluenza virus types (1–4); adenovirus (A-F); rhinovirus; metapneumovirus; coronavirus (NL63, 229E, OC43) and bocavirus (Methods previous described in Cebey-López et al. [4]). No difference was found on the molecular diagnosis yield in our series when using aspirate or swab as sample source.

All investigators were trained in the study protocol for patient recruitment, sample processing and sample storage. The study was performed according to Good Clinical Practice. Written informed consent was obtained from a parent or legal guardian for each subject before study inclusion. The study was approved by the Ethical Committee of Clinical Investigation of Galicia (CEIC ref. 2010/015). The UK cohort study was approved by the St Mary’s Research Ethics Committee (REC 09/H0712/58).

### Clinical data collection

Detailed clinical data on each patient were collected using a secured web-based platform. This included risk factors for LT-ARI (prematurity, immunization status, obesity, diabetes, asthma and previous admissions to hospital), current medications, and family history of asthma or other respiratory diseases. The severity of each respiratory case was ranked as follows: 1) physician criteria (mild, moderate or severe); 2) Wood-Downes scale (0 to 10 points; mild < 3, moderate 4–7, severe > 8); and 3) a newly developed scale -named GENVIP score- (0 to 20 points) that assesses food tolerance, degree of medical intervention needed, respiratory distress, respiratory frequency, apnea, malaise and fever (see S1 Table for further details on this scale). Supplemental oxygen and / or mechanical ventilation requirement during admission were also recorded. Bacterial superinfection diagnosis was established according to the referring physician criteria, based on clinical data, inflammatory markers, and radiological findings, and/or appropriate cultures in sterile sites (e.g blood or cerebrospinal fluid). For the study purposes, cases classified as bacterial co-infections included both cases with and without microbiological confirmation as in our series, cultures were not carried out systematically, but only when a bacterial superinfection was suspected by the referring physician.

### Data analysis

In this analysis, we compared clinical data to the results of pathogen identification in respiratory samples. We performed all the analysis using the R Software, Version 3.0.2 (www.r-project.org). General data were presented as means with 95% confidence intervals (CI). Different statistical models were used to assess the bivariate association between the variables depending on the dependent variable. The relationship between demographic and clinical variables with mono-infection and co-infection was analysed using simple logistic regression. A binary logistic model was used for the binary variables (co-infection status, oxygen requirements, respiratory support needed and PICU admission), linear model for continuous variables (Wood-Downes Score and GENVIP Score), negative binomial regression model for counted data (hospital stay length) and logistic multinomial model for the multinomial variable (respiratory distress status). Multiple regression models were considered using the significant risk factors obtained in the bivariate analysis and sex and age variables. In order to reduce the likelihood of false significant results due to too many statistical comparisons, the Bonferroni multiple test correction were considered. A χ2 test was performed to evaluate the correlation between bacterial superinfection and pneumococcal vaccine. Level of statistical significance was set at 0.05.

## Results

The GENDRES cohort included nasopharyngeal samples from 204 patients with a median age of 16.7 (95% CI: 12.7–20.6) months and a male-to-female sex ratio of 1.7. One patient was excluded due to incomplete clinical data. In the UK a cohort of 97 patients’ data was analyzed, with a median of age of 36.6 (95% CI: 27.6–45.6) months and a male-to-female ratio of 0.94 (S1 Fig). Comparison between both cohorts is shown in Table 1.

**Table 1:** Description of the characteristics of the two cohorts analyzed: the GENDRES cohort and the UK cohort. *P*-value results from the comparison between both cohorts. A *P*-value < 0.05 was considered significant.

Variable	GENDRES cohort n (%)	UK cohort n (%)	*P*-value
Sex (female proportion) ^1^	75 (36.9)	50 (51.5)	0.018
Age (months) ^1^			<0.001^β^
0–12	136 (66.7)	39 (40.2)	
13–24	25 (12.3)	17 (17.5)	
25–48	26 (12.8)	17 (17.5)	
<48	16 (7.9)	24 (24.7)	
Pneumoccocal vaccine^1^	110 (53.9)	57 (64.0)	0.124
Bacterial superinfection^1^	56 (29.5)	53 (54.6)	<0.001^β^
Mono-infection^1^	95 (46.6)	56 (57.7)	0.084
RSV	53 (28.3)	21 (24.7)	
Rhinovirus	17 (9.1)	12 (14.1)	
Co-infection^1^	92 (49.2)	29 (34.1)	0.012
RSV + rhinovirus	23 (12.3)	3 (3.5)	
RSV+ bocavirus	10 (5.3)	5 (5.9)	
PICU admission^1^	38 (29.0)	43 (44.3)	0.024
Respiratory support^1^	30 (14.8)	36 (38.3)	<0.001^β^
Oxygen needed^1^	56 (29.5)	55 (57.9)	<0.001^β^
Hospital stay length^2^*	6 (4, 9)	4 (2, 9)	<0.001^β^

^1^Fisher Exact Test.

^2^Wilcoxon test.

*Median and interquartile range in days.

^β^Significant under Bonferroni correction.

### Viral mono-infection *versus* viral multi-infection

The PCR results were preciously described in Cebey-López et al. [4]. Clinical data, family or past medical history, need of PICU admission or hospital length of stay, and oxygen or respiratory support need, were equivalent in the GENDRES and the UK cohort (Table 2).

**Table 2:** Relationship between demographic and clinical variables with mono-infection and co-infection is shown for both GENDRES and UK cohort. The correlation was analysed using simple logistic regression. Data are presented as OR (95% confidence interval) and *P*-value. The characteristics of the two cohorts analyzed were compared and when P-value results were significant when different: the GENDRES cohort and the UK cohort. *P*-value results from the comparison between both cohorts. A *P*-value < 0.005 was considered significant.

Variable	GENDRES cohort (*n* = 203)					UK-cohort (*n* = 97)				
	GENDRES% (95% CI)	OR (95% CI)	*P*-value	Multiple OR (95% CI)	*P* -value	UK-cohort % (95% CI)	OR (95% CI)	*P* -value	Multiple OR (95% CI)	*P* -value
**Demographic characteristics**										
Sex. Female	36.9	1.287	0.405	1.069	0.838	51.5	1.228	0.657	1.14	0.802
	(30.3, 43.6)	(0.711, 2.340)		(0.564, 2.014)		(41.1, 62.0)	(0.498, 3.086)		(0.41, 3.18)	
Age			^**β**^							
12–24 months	12.3	3.723	0.010	3.173	0.028	17.5	8.937	0.002	8.73	0.002
	(7.8 16.8)	(1.428, 10.955)		(1.177, 9.569)		(9.4, 25.6)	(2.377, 40.192)		(2.3, 39.66)	
24–48 months	12.8	4.189	0.005	3.463	0.018	17.5	1.773	0.378	1.79	0.372
	(8.2, 17.4)	(1.635, 12.201)		(1.290, 10.447)		(9.4, 25.6)	(0.484, 6.373)		(0.49, 6.44)	
> 48 months	7.9	0.621	0.447	0.544	0.339	24.7	0.867	0.836	0.84	0.802
	(4.2, 11.6)	(0.161, 2.015)		(0.139, 1.800)		(15.6, 33.9)	(0.203, 3.261)		(0.19, 3.24)	
**Family history**										
Asthma	39.9	0.753	0.343			n.a.				
	(33.2, 46.6)	(0.417, 1.352)								
Respiratory conditions	15.8	0.824	0.623			n.a.				
	(10.8, 20.9)	(0.375, 1.785)								
**Patient medical history**										
Premature birth	8.5	0.450	0.199			n.a.				
	(4.5, 12.5)	(0.118, 1.443)								
Pneumococcal vaccine	53.9	2.055	0.016	1.550	0.176	64.0	1.990	0.189		
	(47.1, 60.8)	(1.151, 3.709)		(0.821,2.932)		(54.1, 74.0)	(0.734, 5.849)			
Pulmonary conditions	3.5	0.517	0.453			n.a.				
	(0.9, 6.0)	(0.070, 2.718)								
Asthma	11.8	0.943	0.899			n.a				
	(7.4, 16.3)	(0.374, 2.354)								
**Clinical data**										
Bacterial superinfection	29.5	1.396	0.308			54.6	1.319	0.549		
	(23.0, 36.0)	(0.736, 2.667)				(44.2, 65.1)	(0.536, 3.315)			

^β^Significant under Bonferroni correction.

In the GENDRES cohort the presence of rhinovirus as co-pathogen was associated with a significantly increased Wood-Downes score by 1.289 points (95% CI: 0.387, 2.192); *P*-value = 0.006. RSV infection was associated with increased oxygen requirements [OR (95% CI): 3.154 (1.302, 7.966); *P*-value = 0.012] (Table 3). These isolated findings were not replicated in the UK cohort (Table 3).

**Table 3:** Comparison of virus and disease severity of the main cohort considering the virus as single pathogen or as co-infection in the sample. Different statistical models were considered to study the bivariate association between the variables depending on the dependent variable. A binary logistic model was used for the binary variables oxygen needed and respiratory support needed, and a negative binomial regression model for counted data (hospital stay length). Data are presented as OR (confidence interval 95%) and the level of statistical significance was set at 0.05.

*Risk Factor*	*Hospital stay length*		*Oxygen needed*		*Respiratory support*		*Wood Downes score*		*GENVIP score*		*PICU admission*	
	(*n* = 163)		(*n* = 186)		(*n* = 186)		(*n* = 177)		(*n* = 125)		(*n* = 119)	
	OR (95% CI)	*P* -value	OR (95% CI)	*P* -value	OR (95% CI)	*P*–value	Coefficient (95% CI)	*P* -value	Coefficient (95% CI)	*P* -value	OR (95% CI)	*P* -value
**Mono-infected**												
RSV	1.092	0.450	3.154	0.012	3.556	0.122	0.571	0.188	1.384	0.107	1.121	0.846
	(0.868, 1.374)		(1.302, 7.966)		(0.832, 24.487)		(-0.284,1.425)		(-0.307, 3.075)		(0.362, 3.728)	
Rhinovirus	0.785	0.154	0.327	0.042	1.167	0.854	-0.823	0.144	-1.134	0.447	1.410	0.623
	(0.562, 1.094)		(0.109, 0.962)		(0.165, 5.257)		(-1.932, 0.285)		(-4.095, 1.826)		(0.330, 5.410)	
Bocavirus	0.999	0.997	0.435	0.327			-0.187	0.859	-2.610	0.111		
	(0.646, 1.548)		(0.076, 2.482)				(-2.270, 1.897)		(-5.840, 0.619)			
Adenovirus	1.211	0.456					0.859	0.413	1.581	0.624		
	(0.733, 2.019)						(-1.217, 2.935)		(-4.841, 8.002)			
**Co-infected**												
RSV	1.150	0.281	0.938	0.892	1.550	0.456	-0.222	0.646	0.243	0.815	1.426	0.597
	(0.892, 1.481)		(0.361, 2.364)		(0.509, 5.328)		(-1.217, 2.935)		(-1.829, 2.316)		(0.402, 5.887)	
Rhinovirus	1.443	0.003	1.642	0.288	2.921	0.085	1.289	0.006	0.920	0.379	2.169	0.146
	(1.135, 1.836)		(0.658, 4.158)		(0.923, 11.199)		(0.387, 2.192)		(-1.157, 2.997)		(0.104, 1.398)	
Bocavirus	0.697	0.003	1.243	0.642	0.889	0.832	-0.654	0.168	-0.551	0.589	1.174	0.794
	(0.551, 0.883)		(0.499, 3.168)		(0.290, 2.632)		(-1.590, 0.282)		(-2.583, 1.481)		(0.352, 3.910)	
Adenovirus	0.938	0.613	1.000	1.000	0.682	0.515	0.290	0.553	-0.848	0.422	1.426	0.597
	(0.730, 1.204)		(0.396, 2.604)		(0.198, 22.080)		(-0.677, 1.257)		(-2.947, 1.252)		(0.383, 5.305)	

### Bacterial superinfection

**Fig 1:**
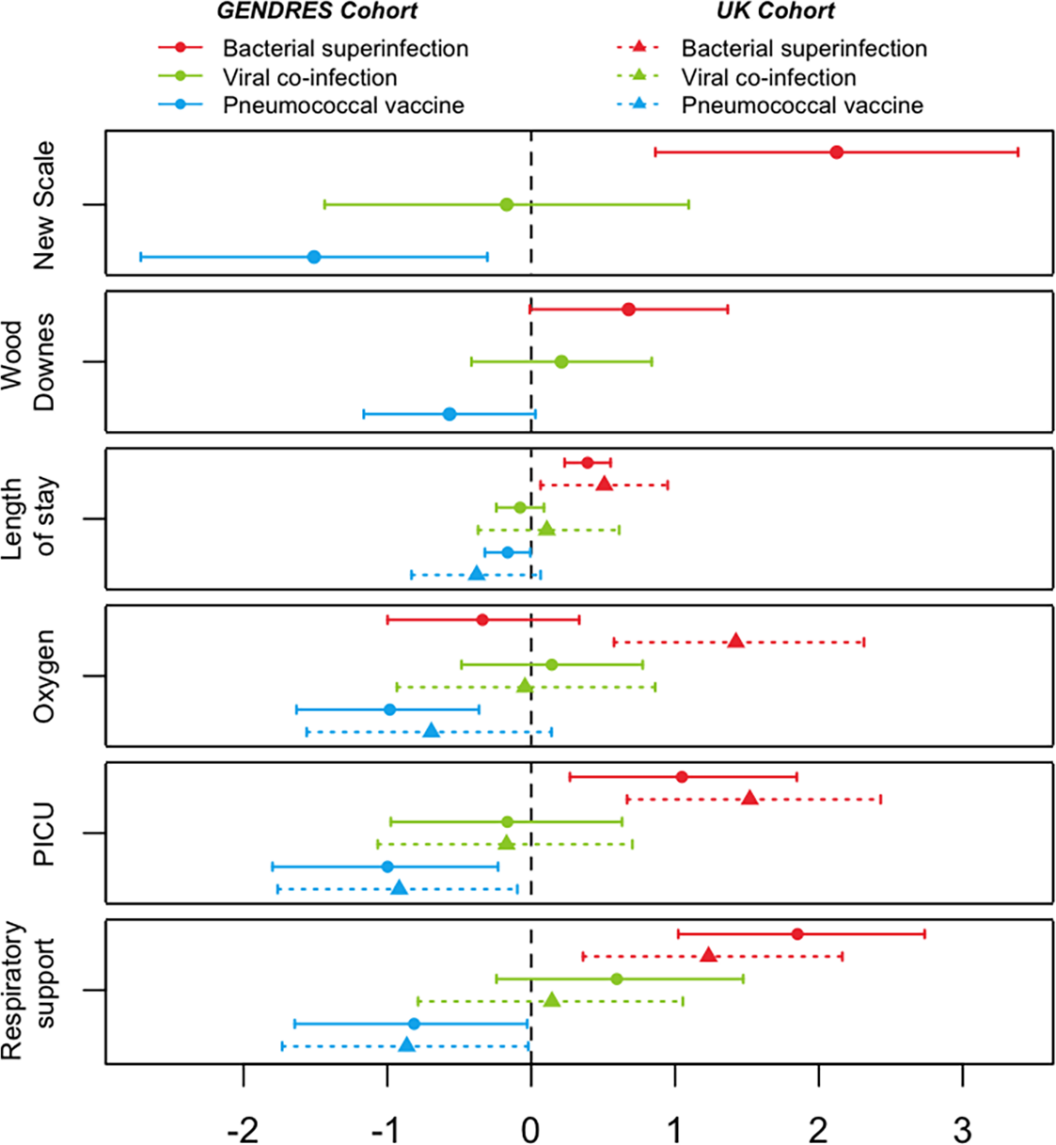
Influence of bacterial superinfection, pneumococcal vaccine and the presence of viral co-infection on disease severity of children with ARI, according to oxygen and respiratory support requirement, clinical scales, hospital stay length and PICU admission. Data are shown as OR (95% CI) for both main cohort and replication cohort. A binary logistic model was used for the binary variables (co-infection status, oxygen requirements, respiratory support needed and PICU admission), linear model for continuous variables (Wood-Downes Score and the GENVIP score) and negative binomial regression model for counted data (number of days since admission).

Children presenting a bacterial superinfection had more severe respiratory distress [OR (95% CI): 4.356 (1.564, 12.128); *P*-value = 0.005] and a higher severity score [2.124 (95% CI: 0.864, 3.385); *P*-value = 0.001]. They were more likely to be admitted to PICU in the GENDRES cohort [OR (95% CI): 2.851 (1.300, 6.252); *P*-value = 0.009] and the UK cohort [5.357 (2.081, 15.085); *P*-value = 0.001]. Children with bacterial co-infection required significantly more respiratory support in both cohorts: discovery cohort [OR (95% CI): 6.368 (2.724, 14.886); *P*-value < 0.001] and replication cohort [OR (95% CI): 3.432 (1.402, 8.404); *P*-value = 0.007], and they had a longer hospital stay in both cohorts: 1.48 days (*P*-value = 0.025) longer stay in GENDRES cohort and 1.87 days (*P*-value = 0.005) in UK cohort, respectively (Fig 1; S3–S8 Tables). In addition, 34.0% of the patients with bacterial infection in the GENDRES cohort received the pneumococcal vaccine and 24.7% did not receive it (*P*-value = 0.213).

### Pneumococcal vaccine

In the GENDRES cohort the pneumococcal vaccine was given to 53.9% (46.9, 61.1) of the patients of whom 43.8% (33.3, 54.2) were mono-infected and 62.7% (52.2, 73.1) were viral co-infected patients [OR (95% CI): 1.550 (0.821, 2.932); *P*-value = 0.176]. Vaccinated patients had lower risk of being admitted to PICU in GENDRES cohort [OR (95% CI): 0.301 (0.116, 0.735); *P*-value = 0.011] and in the UK cohort [OR (95% CI): 0.208 (0.046, 0.776); *P*-value = 0.027] and had less risk of respiratory support requirement in the main cohort [OR (95% CI): 0.324 (0.124, 0.790); *P*-value = 0.016] and in the replication one [OR (95% CI): 0.267 (0.070, 0.901); *P*-value = 0.040]. In the Spanish cohort, patients who received the pneumococcal vaccine received less oxygen support [OR (95% CI): 0.328 (0.162, 0.639); *P*-value = 0.001], and had a lower clinical severity score [-1.499 (95% CI: -2.768, -0.231) points; *P*-value = 0.021] and a lower respiratory distress score [OR (95% CI): 2.917 (1.078, 7.889); *P*-value = 0.035]. These findings were not replicated in the UK cohort (Fig 1; S1–S7 Tables).

## Discussion

Our study revealed that even though multiple viral detection is frequent in hospitalized children with LT-ARI, this association is not related to either disease severity or to any other clinical features studied. PICU admission, disease severity according to different scales, need for respiratory support, and length of hospital stay followed a similar pattern in viral mono- versus co-infected children. Contrariwise, bacterial superinfection increased the severity of the disease course, while pneumococcal vaccination played a protective role.

The detection of multiple coincident viruses in clinical settings is becoming more common since the introduction of molecular based multiplex tests, but the clinical significance of these findings remains unclear and seems to have no impact in disease severity [5]. Both an increase in disease severity in relation to dual infections [6–9] and the absence of this association [10–19] have been reported. Richard et al. [8] found that co-infected children were almost three times more likely to be admitted to the PICU than those with single viral infections. Compared to our study Richard et al. developed a retrospective and monocentric study in which they only considered dual infections, infants and bronchiolitis.

There is contradictory evidence linking disease severity with specific respiratory viruses. A shorter hospital stay has been reported in children with rhinovirus bronchiolitis than with RSV [20]. Rhinovirus and RSV co-infection is reported to increase the risk of severe disease [8] or the bronchiolitis relapse [21, 22]. Other studies did not find significant differences in severity between co-infection and single infection [12, 18, 23, 24]. In our study we did not find increased severity of illness in children with RSV-rhinovirus dual infection. In our series, only RSV as mono-infection increased oxygen requirements, and rhinovirus as a co-infecting pathogen increased the Wood-Downes score in the Spanish cohort, but these isolated findings arising from the multivariate analysis could not be replicated in the UK cohort.

Several studies have reported increased severity with bocavirus (hBoV) co-infections [13, 25–27]; this was not the case in our series (also in agreement with Pen et al. [15]). hBoV was commonly detected in our patients, with no impact in the severity of the illness. As hBoV was detected in alongside other respiratory viruses with an established pathogenic potential, it is possible that hBoV detection reflects asymptomatic persistence or prolonged viral shedding [28].

Bacterial superinfection was the only factor consistently linked to greater severity. Studies of the pandemic influenza indicate that respiratory viruses predispose to bacterial complication and interaction between viruses and bacteria in respiratory infections has been extensively reported in the literature [29], but the underlying mechanisms between viral and bacterial synergism are complex and remain unclear [30]. Common respiratory viral infections, such as influenza or respiratory syncytial virus have been linked to seasonal increases in *Streptococcus pneumoniae* disease [31]. The relationship between bacterial and viral infection is clouded by the low sensitivity of bacterial detection in sterile-site samples by traditional culture methods, and the reliance on non-specific clinical data for the for diagnosis of bacterial co-infection, including inflammatory markers, radiological findings and / or appropriate cultures, resulting in 30% of the cases in the GENDRES cohort and 55% in the UK cohort. Bacterial superinfection increased most measures of severity in both cohorts (PICU admission, respiratory support requirement, GENVIP score, hospital stay length and respiratory distress).

Interestingly, pneumococcal vaccination was revealed as an independent protective factor of disease severity in our patients. Pneumococcal vaccine reduced the severity of viral LT-ARIs through a reduction in oxygen requirement, invasive and non-invasive ventilation, admission to PICU, respiratory distress, and GENVIP score. A reduced incidence of viral alveolar pneumonia has been previously reported after pneumococcal vaccination [31, 32], although there was no demonstrable reduction in the number of confirmed pneumococcal infections. This is likely to reflect the limited sensitivity of culture-proven pneumococcal disease in pneumonia. The protective effect of pneumococcal vaccines found in our study might reinforce the importance of the paradigm of viral-penumococci interaction at nasopharynx level in the pathogenesis and clinical course of the disease [33] Current pneumococcal conjugate vaccines significantly decrease nasopharyngeal carriage of pneumococci [34] and thus reduces the possibility of this viral-pneumococci direct interaction.

One of the limitations of the present study is that our samples were not tested for viral load by quantitative PCR and the viral load of certain viruses–like RSV- has been associated with the co-infection status and the severity [3]. Also, the study did not consider milder or asymptomatic children. In addition, bacterial superinfection rate in our series might be overestimated as diagnosis was accepted as true even without microbiological confirmation, just based on referring physicians’ criteria.

Several studies had shown that viruses can be found in children with no respiratory infections [6, 35], and further research is needed to understand the natural history of respiratory viral carriage and infection. However, our findings were consistent in both independent cohorts very different between them, so this makes the outcomes more robust.

In summary, the severity and course of an acute respiratory episode requiring hospitalization in children did not correlate with the presence of one or more viruses. In contrast, bacterial co-infection was associated with more severe disease, whereas pneumococcal vaccination decreased severity. Future studies are needed to investigate whether particular viruses, or combinations of viruses, influence the risk of bacterial co-infection.

## Supporting Information

S1 Fig Flow chart of study population of the main cohort (GENDRES cohort) and replication cohort (UK-cohort). (JPG)

S1 Table GENVIP score. (DOCX)

S2 Table Demographic characteristics, family and patient medical history, clinical course and principal virus in children with ARI and disease severity, considering respiratory support and oxygen requirement the characteristics that described the severity of the illness of the main cohort. (DOCX)

S3 Table Variables analyzed in children with ARI and disease severity, considering the clinical scales the characteristics that described the severity of the illness of the main cohort. (DOCX)

S4 Table Demographic characteristics, family and patient medical history, clinical course and main virus in children with ARI and disease severity in GENDRES cohort. (DOCX)

S5 Table Children’s with ARI characteristics and moderate and severe respiratory distress. (DOCX)

S6 Table Comparison of virus and disease severity of the replication cohort (UK cohort) considering the virus as single pathogen or as co-infection in the sample. (DOCX)

S7 Table Demographic characteristics, clinical course and principal virus in children with ARI and disease severity, considering respiratory support and oxygen requirement the characteristics that described the severity of the illness of the UK-cohort are presented. (DOCX)

S8 Table Variables analyzed in the UK-cohort children and disease severity according to hospital stay length and PICU admission are shown. (DOCX)
