# Supplementary material for: Does Viral Co-Infection Influence the Severity of Acute Respiratory Infection in Children?
Source: PLoS One. 2016 Apr 20;11(4):e0152481. doi: 10.1371/journal.pone.0152481 (PMC4838299; doi:10.1371/journal.pone.0152481)
Supplement: S1 Table — (DOCX) [file pone.0152481.s002.docx]

**S1 Table**

**CLINICAL SEVERITY SCORE FOR HEALTHY INFANTS WITH RESPIRATORY INFECTIONS**

For each of the 6 items, please indicate the option that better describes the situation of the child. Always consider the worst condition anytime during the whole course of the patient illness. Scoring in each item ranges from 0 to 3 points, and the minimum global scoring is 0, and the maximum is 20 points.

|  |  | **0 points** | **1 points** | **2 points** | **3 points** | **TOTAL POINTS** |
| --- | --- | --- | --- | --- | --- | --- |
| **1** | **Feeding intolerance** | **No** | **Mild**  Decreased appetite and/or isolated vomits with cough. | **Partial**  Frequent vomits with cough, rejected feed but able to tolerate fluids sufficiently to ensure hydration | **Total**  Oral intolerance or absolute rejection of oral feed, not able to guarantee adequate hydration orally. Required nasogastric and/or intravenous fluids |  |
| 2 | **Medical intervention** | **No** | **Basic**  Nasal secretions aspiration, physical examination, trial of nebulised bronchodilators, antipyretics. | **Intermediate**  Oxygen therapy required. Complementary exams were needed (chest X-ray, blood gases, hematimetry…). Mantained nebulised therapy with bronchodilators | **High**  Required respiratory support with positive pressure (either non-invasive in CPAP, BiPAP or high-flow O2; or invasive through endotracheal tube). |  |
| 3 | **Respiratory difficulty** | **No** | **Mild**  Not in basal situation but do not impress of severity. Wheezing only audible with stethoscope, good air entrance. If modified Wood Downes, Wang score or any other respiratory distress score is applied, punctuation reveals mild severity. | **Moderate**  Makes some extra respiratory effort (intercostal and/or tracheosternal retraction). Presented expiratory wheezing audible even without stethoscope, and air entrance may be localized decreased. If modified Wood Downes, Wang score or any other respiratory distress score is applied, punctuation reveals mild severity. | **Severe**  Respiratory effort is obvious. Inspiratory and expiratory wheezing and/or clearly decreased air entry. If modified Wood Downes, Wang score or any other respiratory distress score is applied, punctuation reveals mild severity. |  |
| 4 | **Respiratory frequency** | **Normal**  < 2 m: 40-50 bpm  2-6 m: 35-45 bpm  6-12m:30-40 bpm  12-24m: 25-35 bpm  24-36m: 20-30 bpm | **Mild or occasional tachypnea**  Presented episodes of tachypnea, well tolerated, limited in time by self-resolution or response to secretion aspiration or nebulisation. | **Prolongued or recurrent tachypnea**  Tachypnea persisted or recurred despite secretion aspiration and/or nebulisation with bronchodilators. | **Severe alteration**  Severe and maintained tachypnea. Very superficial and quick breath rate. Normal/low breath rate with obvious increased respiratory effort and/or mental status affected.  Orientative rates of severe tachypnea:  < 2 m: > 70 bpm  2-6 m: > 60 bpm  6-12m: >55 bpm  12-24m: >50 bpm  24-36m: >40 bpm |  |
| 5 | **Apnea** | **No** |  |  | **Yes**  At least one episode of respiratory pause medically documented or strongly suggested through anamnesis. |  |
| 6 | **General Condition** | **Normal** | **Mild**  Not in basal situation, child was mildly uncomfortable but did not impress of severity. Parent are not alarmed. Could wait in the waiting room or even stay at home. | **Moderate**  Patient looks ill, and will need medical exam and eventually further complementary exams and/or therapy. Parent are concern. Not to be waiting in the waiting room. | **Severe**  Agitated, apathetic, lethargic. No need to be physician to be worried. Parent are very concern. Immediate medical evaluation and/or intervention was required |  |
| 7 | **Fever** | **No** | **Yes, mild**  Central Tª <38,5ºC | **Yes, moderate**  Central Tª 38,5-39C | **Yes, severe**  Central >39ºC |  |
|  | | | | **TOTAL SCORE** | |  |
