# Supplementary material for: Does Viral Co-Infection Influence the Severity of Acute Respiratory Infection in Children?
Source: PLoS One. 2016 Apr 20;11(4):e0152481. doi: 10.1371/journal.pone.0152481 (PMC4838299; doi:10.1371/journal.pone.0152481)
Supplement: S2 Table — (DOCX) [file pone.0152481.s003.docx]

| **Variable** | **Respiratory support**  **(n = 203)** | | | | **Oxygen needed**  **(n = 203)** | | | |
| --- | --- | --- | --- | --- | --- | --- | --- | --- |
|  | OR (95% CI) | *P*-value | Multiple OR (95% CI) | *P* -value | OR (95% CI) | *P*-value | Multiple OR (95% CI) | *P* -value |
| **Demographic characteristics** | | | | | | | | |
| Sex (female proportion) | 0.470 (0.191, 1.155) | 0.100 | 0.463 (0.156, 1.216) | 0.136 | 0.766 (0.416, 1.410) | 0.392 | 0.709 (0.370, 1.361) | 0.300 |
| Age |  |  |  |  |  |  |  |  |
| 13 - 24 months |  |  |  |  | 1.217 (0.498, 2.972) | 0.667 | 1.421 (0.557, 3.829) | 0.471 |
| 25 - 48 months |  |  |  |  | 0.649 (0.243, 1.731) | 0.388 | 3.532 (1.257, 11.237) | 0.022 |
| > 48 months |  |  |  |  | 0.983 (0.322, 3.005) | 0.976 | 1.756 (0.556, 6.269) | 0.354 |
| **Family history** | | | | | | | | |
| Asthma | 1.181 (0.539, 2.586) | 0.678 |  |  | 1.500 (0.806, 2.793) | 0.201 |  |  |
| Respiratory conditions | 1.790 (0.695, 4.610) | 0.228 |  |  | 1.158 (0.502, 2.671) | 0.731 |  |  |
| **Patient medical history** | | | | | | | | |
| Premature birth | 2.159 (0.641, 7.269) | 0.214 |  |  | 0.527 (0.186, 1.492) | 0.228 |  |  |
| Pulmonary conditions | 2.489 (0.460, 13.481) | 0.290 |  |  | 0.325 (0.071, 1.499) | 0.325 |  |  |
| Asthma | 1.177 (0.372, 3.721) | 0.782 |  |  | 2.458 (0.804, 7.518) | 0.115 |  |  |
| Pneumococcal vaccine | **0.443 (0.199, 0.987)** | **0.046** | **0.324 (0.124, 0.790)** | **0.016** | **0.374 (0.199, 0.704)** | **0.002^♭♯^** | **0.328 (0.163, 0.639)** | **0.001** |
| **Clinical data** | | | | | | | | |
| Bacterial superinfection | **6.368 (2.724, 14.886)** | **<0.001^♭♯^** | **7.484 (3.113, 19.254)** | **<0.001** | 0.713 (0.367,1.383) | 0.316 |  |  |
| Co-infection | 1.859 (0.796, 4.346) | 0.152 |  |  | 1.119 (0.597, 2.096) | 0.726 |  |  |
| **Virus** | | | | | | | | |
| RSV | 1.630 (0.732, 3.629) | 0.231 |  |  | 1.831 (1.003, 3.340) | 0.049 | 2.040 (1.054, 3.998) | 0.035 |
| Rhinovirus | 1.981 (0.902, 4.351) | 0.809 |  |  | 0.979 (0.520, 1.840) | 0.947 |  |  |
| Bocavirus | 0.848 (0.302, 2.382) | 0.755 |  |  | 1.123 (0.553, 2.280) | 0.749 |  |  |
| Adenovirus | 0.980 (0.392, 2448) | 0.965 |  |  | 1.325 (0.600, 3.927) | 0.486 |  |  |

- **S2 Table:** Demographic characteristics, family and patient medical history, clinical course and principal virus in children with ARI and disease severity, considering respiratory support and oxygen requirement the characteristics that described the severity of the illness of the main cohort. A binary logistic model was used. Data are presented as OR (95% confidence interval) and the level of statistical significance was set at 0.05. Two multiple test correction were considered: Bonferroni correction and FDR.
