# Supplementary material for: Does Viral Co-Infection Influence the Severity of Acute Respiratory Infection in Children?
Source: PLoS One. 2016 Apr 20;11(4):e0152481. doi: 10.1371/journal.pone.0152481 (PMC4838299; doi:10.1371/journal.pone.0152481)
Supplement: S3 Table — (DOCX) [file pone.0152481.s004.docx]

- **S3 Table:** Variables analyzed in children with ARI and disease severity, considering the clinical scales the characteristics that described the severity of the illness of the main cohort. A linear model for continuous variables was used and the level of statistical significance was set at 0.05. Two multiple test correction were considered: Bonferroni correction and FDR.

| **Variable** | **GENVIP score**  **(n = 136)** | | | | **Wood Downes score**  **(n = 192)** | | | | |
| --- | --- | --- | --- | --- | --- | --- | --- | --- | --- |
|  | Coefficient  (95% CI) | *P*-value | Múltiple coefficient  (95% CI) | *P*-value | Coefficient  (95% CI) | *P*-value | Múltiple coefficient  (95% CI) | *P*-value | |
| **Demographic characteristics** | | | | | | | | |  |
| Sex (female proportion) | -0.749 (-2.006, 0.508) | 0.241 | -0.469 (-1.710, 0.774) | 0.457 | -0.158 (-0.783, 0.467) | 0.619 | -0.080 (-0.713, 0.554) | 0.804 | |
| Age |  |  |  |  |  |  |  |  | |
| 13 - 24 months | -1.223 (- 2.990, 0.545) | 0.174 | -1.067 (-2.903, 0.769) | 0.252 | -0.454 (-1.380, 0.472) | 0.335 | -0.429 (-1.375, 0.497) | 0.356 | |
| 25 - 48 months | -1.744 (-4.087, 0.600) | 0.144 | 0.032 (-2.410, 2.474) | 0.980 | -0.707 (-1.618, 0.203) | 0.127 | -0.694 (-1.613, 0.224) | 0.138 | |
| > 48 months | 1.023 (-1.946, 3.994) | 0.497 | 3.274 (-0.148, 6.696) | 0.061 | -0.287 (-1.391, 0.816) | 0.608 | -0.283 (-1.390, 0.825) | 0.615 | |
| **Family history** | | | | | | | | |  |
| Asthma | -0.686 (-0.589, 1.960) | 0.289 |  |  | 0.410 (-0.196, 1.017) | 0.184 |  |  | |
| Respiratory conditions | 1.583 (0.068, 3.099) | 0.041 | 1.168 (-0.391, 2.726) | 0.141 | 0.002 (-0.807, 0.811) | 0.995 |  |  | |
| **Patient medical history** | | | | | | | | |  |
| Premature birth | 1.131 (-1.337, 3.599) | 0.366 |  |  | 0.147 (-1.052, 1.347) | 0.809 |  |  | |
| Pulmonary conditions | -1.008 (-4.259, 2.244) | 0.541 |  |  | -0.471 (-2.069, 1.127) | 0.561 |  |  | |
| Asthma | -0.213 (-2.165, 1.739) | 0.829 |  |  | 0.298 (-0.612, 1.207) | 0.519 |  |  | |
| Pneumococcal vaccine | **-1.510 (-2.714, -0.305)** | **0.014** | **-1.499 (-2.768, -0.231)** | **0.021** | -0.568 (-1.164, 0.029) | 0.062 |  |  | |
| **Clinical data** | | | | | | | | |  |
| Bacterial superinfection | **2.124 (0.864, 3.385)** | **0.001^♭♯^** | **1.988 (0.737, 3.238)** | **0.002** | 0.678 (-0.010, 1.366) | 0.053 |  |  | |
| Co-infection | -0.095 (-1.360, 1.171) | 0.882 |  |  | 0.155 (-0.472, 0.783) | 0.626 |  |  | |
| **Virus** | | | | | | | | |  |
| RSV | 0.624 (-0.618, 1.865) | 0.322 |  |  | -0.006 (-0.610, 0.597) | 0.984 |  |  | |
| Rhinovirus | 0.129 (-1.178, 1.426) | 0.845 |  |  | 0.355 (-0.287, 0.997) | 0.277 |  |  | |
| Bocavirus | -0.881 (-2.331, 0.569) | 0.232 |  |  | -0.432 (-1.152, 0.287) | 0.237 |  |  | |
| Adenovirus | -0.573 (-2.231, 1.085) | 0.495 |  |  | 0.235 (-0.535, 1.005) | 0.548 |  |  | |
