# Supplementary material for: Does Viral Co-Infection Influence the Severity of Acute Respiratory Infection in Children?
Source: PLoS One. 2016 Apr 20;11(4):e0152481. doi: 10.1371/journal.pone.0152481 (PMC4838299; doi:10.1371/journal.pone.0152481)
Supplement: S4 Table — (DOCX) [file pone.0152481.s005.docx]

- **S4 Table:** Demographic characteristics, family and patient medical history, clinical course and main virus in children with ARI and disease severity in GENDRES cohort. A binary logistic model was used for the binary variable (PICU admission) and a negative binomial regression model for counted data (hospital stay length). Data are presented as OR (confidence interval 95%) and the level of statistical significance was set at 0.05. Two multiple test correction were considered: Bonferroni correction and FDR.

| **Variable** | **Hospital stay length**  **(n = 180)** | | | | **PICU admission**  **(n = 131)** | | | |
| --- | --- | --- | --- | --- | --- | --- | --- | --- |
|  | OR (95% CI) | *P*-value | Multiple OR (95% CI) | *P*-value | OR (95% CI) | *P*-value | Multiple OR (95% CI) | *P*-value |
| **Demographic characteristics** | | | | | | | | |
| Sex (female proportion) | 0.954 (0.809, 1.125) | 0.578 | 1.010 (0.864, 1.181) | 0.901 | 0.777 (0.342, 1.766) | 0.546 | 0.901 (0.343, 2.266) | 0.827 |
| Age |  |  |  |  |  |  |  |  |
| 13 - 24 months | 0.863 (0.675, 1.101) | 0.236 | 0.944 (0.737, 1.206) | 0.643 | 0.645 (0.196, 2.118) | 0.470 | 0.858 (0.170, 3.417) | 0.836 |
| 25 - 48 months | 0.650 (0.502, 0.839) | <0.001 | 0.690 (0.515, 0.918) | 0.011 | 0.323 (0.038, 2.735) | 0.300 | 0.938 (0.046, 6.839) | 0.956 |
| > 48 months | 1.030 (0774, 1.374) | 0.838 | 1.152 (0.833, 1.589) | 0.393 | 2.258 (0.304, 16.770) | 0.426 | 2.529 (0.280, 23.037) | 0.378 |
| **Family history** | | | | | | | | |
| Asthma | 0.869 (0.737,1.024) | 0.094 |  |  | 1.688 (0.773, 3689) | 0.189 |  |  |
| Respiratory conditions | 1.083 (0.869, 1.350) | 0.480 |  |  | 0.941 (0.357, 2.477) | 0.902 |  |  |
| **Patient medical history** | | | | | | | | |
| Premature birth | 1.340 (0.995, 1.815) | 0.556 |  |  | 1.161 (0.333, 4.047) | 0.814 |  |  |
| Pulmonary conditions | 1.170 (0.787, 1.752) | 0.440 |  |  | 1.648 (0.264, 10.281) | 0.593 |  |  |
| Asthma | 0.875 (0.679, 1.128) | 0.303 |  |  | 1.053 (0.258, 4.307) | 0.943 |  |  |
| Pneumococcal vaccine | 0.849 (0.725, 0.994) | 0.042 | 0.874 (0.747, 1.022) | 0.091 | **0.368 (0.169, 0.804)** | **0.012** | **0.301 (0.116, 0.735)** | **0.011** |
| **Clinical data** | | | | | | | | |
| Bacterial superinfection | **1.480 (1.261, 1.737)** | **<0.001^♭♯^** | **1.468 (1.257, 1.715)** | **<0.001** | **2.851 (1.300, 6.252)** | **0.009** | **3.342 (1.438, 8.093)** | **0.006** |
| Co-infection | 0.914 (0.775, 1.078) | 0.287 |  |  | 0.847 (0.381, 1.885) | 0.684 |  |  |
| **Virus** | | | | | | | | |
| RSV | 1.095 (0.933, 1.286) | 0.266 |  |  | 1.133 (0.520, 2.468) | 0.754 |  |  |
| Rhinovirus | 1.071 (0.905, 1.268) | 0.425 |  |  | 1.514 (0.689, 3.326) | 0.302 |  |  |
| Bocavirus | 0.769 (0.634, 0.931) | 0.007 | 0.904 (0.741, 1.101) | 0.316 | 0.643 (0.237, 1.744) | 0.386 |  |  |
| Adenovirus | 0.930 (0.757, 1.143) | 0.490 |  |  | 0.855 (0.285, 2.566) | 0.780 |  |  |
