# Supplementary material for: Does Viral Co-Infection Influence the Severity of Acute Respiratory Infection in Children?
Source: PLoS One. 2016 Apr 20;11(4):e0152481. doi: 10.1371/journal.pone.0152481 (PMC4838299; doi:10.1371/journal.pone.0152481)
Supplement: S5 Table — (DOCX) [file pone.0152481.s006.docx]

- **S5 Table:** Children’s with ARI characteristics and moderate and severe respiratory distress. A logistic multinomial model was used and mild status was fixed as category of reference. Level of statistical significance was set at 0.05.

| **Respiratory distress** | | | | |
| --- | --- | --- | --- | --- |
| **Variable** | **Moderate** | | **Severe** | |
|  | Coefficient (95% CI) | *P*-value | Coefficient (95% CI) | *P*-value |
| **Demographic characteristics** | | | | |
| Sex (female proportion) | 1.060 (0.557, 2.018) | 0.860 | 0.693 (0.248, 1.933) | 0.483 |
| **Family history** | | | | |
| Asthma | 2.274 (0.467, 3.473) | 0.636 | 1.262 (0.289, 5.517 | 0.757 |
| Respiratory conditions | 1.000 (0.419, 2.385) | 1.000 | 1.462 (0.434, 4.923) | 0.540 |
| **Patient medical history** | | | | |
| Premature birth | 0.666 (0.201, 2.205) | 0.666 | 2.063 (0.500, 8.514) | 0.317 |
| Pulmonary conditions | 0.237 (0.042, 1.334) | 0.102 | 0.598 (0.063, 5.642) | 0.598 |
| Asthma | 1.274 (0.467, 3.473) | 0.636 | 1.262 (0.289, 5.517) | 0.757 |
| Pneumococcal vaccine | 1.212 (0.644, 2.280) | 0.552 | 2.917 (1.078, 7.889) | 0.035 |
| **Clinical data** | | | | |
| Bacterial superinfection | 0.877 (0.420, 1.831) | 0.727 | 4.356 (1.564, 12.128) | 0.005 |
| Co-infection | 0.915 (0.477, 1.756) | 0.790 | 1.615 (0.570, 4.578) | 0.367 |
| **Virus** | | | | |
| RSV | 0.858 (0.459, 1.604) | 0.632 | 1.406 (0.532, 3.718) | 0.492 |
| Rhinovirus | 1.251 (0.634, 2.469) | 0.519 | 1.765 (0.657, 4.739) | 0.260 |
| Bocavirus | 1.036 (0.498, 2.152) | 0.925 | 0.643 (0.188, 2.199) | 0.481 |
| Adenovirus | 1.404 (0.606, 3.248) | 0.429 | 1.111 (0.307, 4.024) | 0.873 |
