# Supplementary material for: Does Viral Co-Infection Influence the Severity of Acute Respiratory Infection in Children?
Source: PLoS One. 2016 Apr 20;11(4):e0152481. doi: 10.1371/journal.pone.0152481 (PMC4838299; doi:10.1371/journal.pone.0152481)
Supplement: S6 Table — (DOCX) [file pone.0152481.s007.docx]

- **S6 Table:** Comparison of virus and disease severity of the replication cohort (UK cohort) considering the virus as single pathogen or as co-infection in the sample. Different statistical models were considered to study the bivariate association between the variables depending on the dependent variable. A binary logistic model was used for the binary variables oxygen needed and respiratory support needed, and a negative binomial regression model for counted data (hospital stay length). Data are presented as OR (confidence interval 95%) and the level of statistical significance was set at 0.05.

| **Variable** | ***PICU admission***  ***(n = 97)^b^*** | | ***Respiratory support***  ***(n = 94)^b^*** | | ***Oxygen needed***  ***(n = 95)^b^*** | | ***Hospital stay length***  ***(n = 94)^a^*** | |
| --- | --- | --- | --- | --- | --- | --- | --- | --- |
|  | OR (95% CI) | *P*-value | OR (95% CI) | *P*-value | OR (95% CI) | *P*-value | OR (95% CI) | *P*-value |
| **Mono-infected** |  |  |  |  |  |  |  |  |
| RSV | 1.938 (0.658, 5.877) | 0.233 | 1.385 (0.449, 4.264) | 0.568 | 3.200 (1.000, 11.614) | 0.059 | 0.789 (0.430, 1.478) | 0.449 |
| Rhinovirus | 2.132 (0.536, 9.330) | 0.287 | 2.000 (0.487, 8.270) | 0.327 | 7.200 (1.203, 138.501) | 0.072 | 0.573 (0.264, 1.375) | 0.181 |
| Bocavirus | 0.389 (0.019, 3.265) | 0.427 | 0.561 (0.027, 4.745) | 0.628 | 0.594 (0.067, 5.279) | 0.617 | 0.822 (0.290, 3.122) | 0.739 |
| Adenovirus | 2.609 (0.236, 58.189) | 0.445 | 1.789 (0.068, 47.003) | 0.687 | 0.606 (0.023, 15.898) | 0.728 | 2.224 (0.750, 10.046) | 0.210 |
| **Co-infected** |  |  |  |  |  |  |  |  |
| RSV | 0.804 (0.174, 3.574) | 0.774 | 0.444 (0.085, 2.093) | 0.313 | 1.400 (0.310, 6.604) | 0.662 | 0.667 (0.327, 1.382) | 0.267 |
| Rhinovirus | 0.635 (0.136, 2.803) | 0.551 | 0.833 (0.173, 3.916) | 0.816 | 1.400 (0.310, 6.604) | 0.662 | 1.324 (0.649, 2.724) | 0.440 |
| Bocavirus | 0.700 (0.153, 3.123) | 0.638 | 1.200 (0.255, 5.791) | 0.816 | 0.389 (0.076, 1.779) | 0.234 | 0.523 (0.260, 1.035) | 0.064 |
| Adenovirus | 0.218 (0.010, 1.640) | 0.194 | 0.220 (0.011, 1.683) | 0.199 | 0.286 (0.034, 1.804) | 0.198 | 0.697 (0.301, 1.788) | 0.423 |
