# Supplementary material for: Does Viral Co-Infection Influence the Severity of Acute Respiratory Infection in Children?
Source: PLoS One. 2016 Apr 20;11(4):e0152481. doi: 10.1371/journal.pone.0152481 (PMC4838299; doi:10.1371/journal.pone.0152481)
Supplement: S7 Table — (DOCX) [file pone.0152481.s008.docx]

- **S7 Table:** Demographic characteristics, clinical course and main virus in children with ARI and disease severity, considering respiratory support and oxygen requirement the characteristics that described the severity of the illness of the UK-cohort are presented. A binary logistic model was used. Data are presented as OR (95% confidence interval) and the level of statistical significance was set at 0.05.

| **Variable** | **Oxygen needed**  **(n = 95)** | | | | **Respiratory support**  **(n = 94)** | | | |
| --- | --- | --- | --- | --- | --- | --- | --- | --- |
|  | OR (95% CI) | *P*-value | Multiple OR (95% CI) | *P*-value | OR (95% CI) | *P*-value | Multiple OR (95% CI) | *P*-value |
| Sex (female proportion) | 0.734 (0.321, 1.661) | 0.458 | 0.860 (0.338, 2.190) | 0.749 | 1.197 (0.521, 2.772) | 0.671 | 1.268 (0.478, 3.403) | 0.633 |
| Age |  |  |  |  |  |  |  |  |
| 13 - 24 months | 0.179 (0.047, 0.606) | 0.008 | 0.187 (0.045, 0.692) | 0.015 | 0.238 (0.048, 0.887) | 0.046 | 0.327 (0.059, 1.433) | 0.158 |
| 25 - 48 months | 0.720 (0.2145 2.522) | 0.596 | 0.714 (0.187, 2.816) | 0.623 | 0.396 (0.109, 1.286) | 0.136 | 0.593 (0.142, 2.283) | 0.454 |
| > 48 months | 0.360 (0.120, 1.045) | 0.063 | 0.455 (0.129, 1.547) | 0.211 | 0.507 (0.169, 1.443) | 0.210 | 0.251 (0.055, 0.967) | 0.055 |
| Pneumoccocal vaccine | 0.543 (0.215,1.320) | 0.184 |  |  | 0.372 (0.147, 0.913) | 0.032 | 0.267 (0.070, 0.901) | 0.040 |
| Bacterial superinfection | 4.151 (1.778, 10.112) | 0.001 | 3.842 (1.541, 10.058) | 0.005 | 3.432 (1.434, 8.699) | 0.007 | 3.218 (1.230, 8.898) | 0.020 |
| Co-infection | 0.824 (0.326, 2.100) | 0.681 |  |  | 1.203 (0.462, 3.088) | 0.701 |  |  |
| Virus |  |  |  |  |  |  |  |  |
| RSV | 2.500 (1.047,6.297) | 0.044 | 2.295 (0.780, 7.204) | 0.138 | 0.925 (0.385, 2.181) | 0.859 |  |  |
| Rhinovirus | 2.535 (0.935, 7.701) | 0.079 |  |  | 1.331 (0.503, 3.459) | 0.557 |  |  |
| Bocavirus | 0.587 (0.210, 1.620) | 0.302 |  |  | 1.031 (0.345, 2.925) | 0.954 |  |  |
| Influenza | 0.462 (0.174, 1.190) | 0.112 |  |  | 0.692 (0.239, 1.858) | 0.476 |  |  |
