# Supplementary material for: Does Viral Co-Infection Influence the Severity of Acute Respiratory Infection in Children?
Source: PLoS One. 2016 Apr 20;11(4):e0152481. doi: 10.1371/journal.pone.0152481 (PMC4838299; doi:10.1371/journal.pone.0152481)
Supplement: S8 Table — (DOCX) [file pone.0152481.s009.docx]

| **Variable** | **PICU admission**  **(n=97)** | | | | **Hospital stay length**  **(n=94)** | | | |
| --- | --- | --- | --- | --- | --- | --- | --- | --- |
|  | OR (95% CI) | *P*-value | Multiple OR (95% CI) | *P*-value | OR (95% CI) | *P*-value | Multiple OR (95% CI) | *P*-value |
| Sex (female proportion) | 1.150 (0.515, 2.579) | 0.733 | 1.396 (0.499,4.014) | 0.526 | 1.228 (0.784, 1.921) | 0.368 | 1.481 (0.952, 2.311) | 0.073 |
| Age |  |  |  |  |  |  |  |  |
| 13 - 24 months | 0.120 (0.024, 0.442) | 0.003 | 0.114 (0.018, 0.533) | 0.020 | 0.524 (0.280, 1.011) | 0.047 | 0.526 (0.284, 1.000) | 0.046 |
| 25 - 48 months | 0.233 (0.063, 0.765) | 0.021 | 0.239 (0.051, 0.982) | 0.055 | 1.367 (0.753, 2.576) | 0.315 | 1.409 (0.790, 2.597) | 0.254 |
| > 48 months | 0.400 (0.137, 1.121) | 0.085 | 0.127 (0.022, 0.551) | 0.010 | 0.935 (0.540, 1.645) | 0.813 | 0.837 (0.487,1.455) | 0.519 |
| Pneumoccocal vaccine | 0.324 (0.129, 0.786) | 0.014 | **0.208 (0.046, 0.776)** | **0.027** | 0.755 (0.461, 1.216) | 0.255 |  |  |
| Bacterial superinfection | 4.571 (1.947, 11.350) | 0.001 | **5.864 (2.122, 18.063)** | **0.001** | 1.663 (1.068, 2.583) | 0.024 | **1.865 (1.198, 2.902)** | **0.005** |
| Co-infection | 0.875 (0.348, 2.162) | 0.774 |  |  | 1.086 (0.672, 1.789) | 0.741 |  |  |
| Virus |  |  |  |  |  |  |  |  |
| RSV | 1.308 (0.567, 3.023) | 0.528 |  |  | 0.783 (0.493, 1.265) | 0.308 |  |  |
| Rhinovirus | 1.084 (0.424, 2.743) | 0.864 |  |  | 0.979 (0.586, 1.699) | 0.937 |  |  |
| Bocavirus | 0.613 (0.210, 1.668) | 0.348 |  |  | 0.743 (0.436, 1.320) | 0.290 |  |  |
| Influenza | 0.594 (0.216, 1.541) | 0.294 |  |  | 1.623 (0.994, 2746) | 0.061 |  |  |

- **S8 Table:** Variables analyzed in the UK-cohort children and disease severity according to hospital stay length and PICU admission are shown. A binary logistic model was used for the binary variable (PICU admission) and a negative binomial regression model for counted data (hospital stay length). Data are presented as OR (confidence interval 95%) and the level of statistical significance was set at 0.05.
